# Editorial Note: Aquaporin expression contributes to human transurothelial permeability in vitro and is modulated by NaCl

**DOI:** 10.1371/journal.pone.0359135

**Published:** 2026-09-25

**Authors:** The PLOS One Editors

The *PLOS One* Editors issue this Editorial Note to inform readers that concerns have been raised about results presented in Fig 1a and Fig 3b of this article [1].
